# Unveiling the Chameleon: A Case Report on Acute Intermittent Porphyria

**DOI:** 10.7759/cureus.56222

**Published:** 2024-03-15

**Authors:** Manish Shrestha, Shefali Amin, Christopher Reggio, Arpan Pokhrel, Salina Munankami, Jakob Nypaver, Riju Gupta, Anthony Donato

**Affiliations:** 1 Internal Medicine, Tower Health Medical Group, Reading, USA; 2 General Medicine, Kathmandu Medical College, Kathmandu, NPL; 3 Medicine, Trauma Services, Hematology/Oncology, Drexel University College of Medicine, Philadelphia, USA

**Keywords:** hemin, porphyrin, chronic abdominal pain, abdominal pain, acute intermitent porphyria

## Abstract

Acute intermittent porphyria (AIP) is a rare autosomal dominant metabolic disorder with low penetrance, often presenting with a broad spectrum of clinical manifestations. Acute neurovisceral attacks commonly occur in young women, mimicking signs and symptoms of other medical and psychiatric conditions, thus delaying the diagnosis. We present the case of an 18-year-old female college student with recurrent hospitalizations for intractable abdominal pain, now again with pain and new subjective hematuria. The patient had previously undergone an endoscopy/colonoscopy with negative biopsies and serologies for acute pathology, including celiac disease. Celiac studies were repeated, given the possibility of inadvertent gluten exposure before the onset of the latest symptoms, but were negative. Basic labs and repeat imaging, including contrast-enhanced CT, MRI, and magnetic resonance (MR) enterography of the abdomen, continued to be unremarkable, and the patient’s symptoms were felt to be functional in etiology. The patient’s urinalysis was normal, and pregnancy was also ruled out. The patient continued to have pain despite receiving opiate analgesics, thus prompting a psychiatry consultation. She was diagnosed with acute adjustment disorder with anxiety and was started on hydroxyzine. Due to persistent symptoms, serum and urine samples were sent, revealing low levels of porphobilinogen deaminase (PBGD) and hydroxymethylbilane synthase (HMBS) gene mutation, confirming the diagnosis of AIP. She was treated with oral glucose and outpatient IV hemin infusions with the resolution of symptoms. AIP presents a nonspecific and highly variable clinical picture, often making it a challenging diagnosis due to such a broad differential. While our patient was thought to have acute adjustment disorder due to an unremarkable initial workup, further testing revealed otherwise. This case demonstrates how clinicians must have a high suspicion of AIP when caring for young females, manifesting with neurovisceral and psychiatric signs and symptoms. Timely diagnosis improves a patient’s quality of life and can decrease overutilization of healthcare resources.

## Introduction

Acute intermittent porphyria (AIP) is a rare metabolic disorder with a prevalence of one to two per 100,000 caused by deficiencies in the enzymes of the heme biosynthesis pathway that lead to a build-up of porphyrins or porphyrin precursors [1,2]. It affects women to a greater degree than men, with a ratio of 1.5 and 2 to 1 [1,3]. This leads to an accumulation of porphobilinogen (PBG) and δ-aminolevulinic acid (ALA), substances that can have neurotoxic effects [1]. Diagnosing AIP is often challenging and delayed due to its vague symptoms that resemble other conditions in the differential for acute encephalopathy, acute abdominal pain, and nonspecific psychiatric symptoms [2]. AIP is important to diagnose early as it may lead to acute and potentially life-threatening manifestations [2,3]. We present a case of AIP in a young female with multiple hospitalizations for undiagnosed abdominal pain.

## Case presentation

An 18-year-old female, with a past medical history of exercise-induced asthma, presented to the hospital with intractable abdominal pain. She has a history of recurrent abdominal pain over the past few months and was admitted to the hospital two months prior for abdominal pain. During that hospitalization, she was evaluated with endoscopy and colonoscopy, which were normal, with negative biopsies for Helicobacter pylori and celiac disease. Tissue transglutaminase IgA was also negative. However, she believed her symptoms may be related to celiac disease, so she was following a gluten-free diet at the time of these tests. She admitted to being under stress during this flare due to final examinations in college. Her symptoms were felt to be functional in etiology, and she was discharged home with a plan for outpatient follow-up.

When she arrived at the hospital for the present admission, she stated her symptoms began a few days ago with crampy, diffuse abdominal pain. This slowly progressed to constant pain associated with nausea, vomiting, and decreased oral intake. She noted intermittent tingling in her fingertips over the past few months, which has become more frequent with the current attack. She admitted to intermittent episodes of hematuria during this time, which was not present during her previous abdominal pain flares. She was not menstruating but noted similar abdominal pain preceding her menses the months prior. She denied fevers, chest pain, dyspnea, headaches, bowel changes, or leg swelling. She denied any changes in her gluten-free diet but was concerned about the possibility of inadvertent gluten exposure at her college cafeteria. She stated she was under additional stress during this presentation due to ongoing college exams. In the emergency room, she was noted to have a temperature of 98.2°F, blood pressure of 126/73 mmHg, heart rate of 85 beats per minute, respiratory rate of 18 breaths per minute, and 100% oxygen saturation on room air. Physical examination showed the patient to be uncomfortable with a non-distended abdomen with hypoactive bowel sounds that were diffusely tender to palpation in all four quadrants without rebound, guarding, or rigidity. Admission laboratory values are listed in Table 1, which were remarkable only for mild normocytic anemia with hemoglobin of 12.9 mg/dL and mild anion gap acidosis. Urine test results are listed in Table 2, which were generally unremarkable, except for elevated urobiligen and urine protein. Abdominal X-ray was negative for obstruction or stool burden. CT abdomen and pelvis without contrast were negative. She was given intravenous ketorolac 15 mg three times a day as needed and pantoprazole 40 mg twice a day without improvement in symptoms. Intravenous morphine 4 mg three times a day did provide mild pain relief, but she remained uncomfortable and was admitted to the hospital for further evaluation and management. Celiac studies were repeated, given the possibility of inadvertent gluten exposure, but were negative. As previous endoscopic workups, including biopsies, were nonspecific, MRI enterography was completed to evaluate for small bowel disease, such as inflammatory bowel disease. However, it returned negative. The patient continued to have pain despite receiving opiate analgesics. Given the constellation of vague abdominal pain, neuropsychiatric symptoms, and intermittent dark urine, rarer disorders were considered, including AIP, so porphyria testing was sent on hospital day four. Psychiatry consultation was obtained for concern of a psychosomatic disorder. She was diagnosed with acute adjustment disorder with anxiety. She was started on hydroxyzine 25 mg three times a day as needed, as well as nightly amitriptyline 25 mg, to help with functional bowel symptoms and discharged home with this regimen on hospital day six. 

**Table 1:** Admission laboratory values

Laboratory Tests	Lab Values	Reference Range
Sodium (mmol/L)	139	136-145
Potassium (mmol/L)	4.2	3.5-5.1
Chloride (mmol/L)	103	98-107
CO_2_ (mmol/L)	22.5	21-31
Glucose (mg/dL)	94	70-99
Blood urea nitrogen (mg/dL)	23	7-25
Creatinine (mg/dL)	0.99	0.6-1.3
Calcium (mg/dL)	9.6	8.6-10.3
Anion Gap (mmol/L)	14	5-12
Albumin (g/dL)	4.3	3.5-5.7
Total protein (g/dL)	6.6	6.4-8.9
Lipase (IU/L)	9	11-82
Alkaline phosphatase (IU/L)	88	34-104
Aspartate aminotransferase (IU/L)	30	13-39
Alanine aminotransferase (IU/L)	25	7-52
Direct bilirubin (mg/dL)	0.1	0.0-0.2
Total bilirubin (mg/dL)	0.9	0.3-1.0
Lactic acid (mmol/L)	1.3	0.6-1.4
White blood cell count (x10^3^/µL)	5.8	4.8-10.8
Red blood cell count (x10^6^/µL)	4.37	4.5-6.1
Hemoglobin (g/dL)	12.9	14-17.5
Hematocrit (%)	38.4	39-53
Platelet count (x10^3^/µL)	180	130-400
Tissue transglutaminase, IgA (U/mL)	<1.2	<4.0

**Table 2:** Urine test results

Urine Tests	Lab Values	Reference Range
Color	Yellow	Yellow, dark yellow, light yellow
Appearance	Clear	Clear
Glucose	Negative	Negative
Bilirubin	Moderate	Negative
Ketones (mg/dL)	100	Negative
Specific gravity	1.040	1.005-1.028
Blood	Negative	Negative
pH	6.0	5.0-8.0
Protein (mg/dL)	70	Negative
Urobilinogen (mg/dL)	4	0.2-1.0
Nitrite	Negative	Negative
Leukocytes	Negative	Negative
Pregnancy test	Negative	Negative

Eleven days after her initial presentation, porphyria results returned with elevated urine (Table 3) and serum porphyrins (Table 4), consistent with AIP. She was evaluated by a hematology outpatient the week following hospital discharge and given a high-dose oral glucose load of 400 mg daily and a course of hemin infusions of 4 mg/kg daily for four days with improvement in symptoms. Further testing revealed diminished porphobilinogen deaminase (PBGD) activity (value: 5.7 nmol/L/sec; normal: > 7 nmol/L/sec) and mutated hydroxymethylbilane synthase (HMBS) gene, thereby confirming the diagnosis of AIP. Over the next couple of months, she continued to have frequent monthly attacks requiring hemin infusions and carbohydrate loading as an outpatient. Her neurovisceral symptoms were thought to be precipitated by ovulation with her menstrual cycles, as the penetrance of AIP is variable but likely more prevalent in females due to progesterone. She was concerned about her significant 11 kg weight gain within these four months due to carbohydrate loading, a known side effect. To better control her symptoms and side effects, she was started on prophylactic pre-menstrual hemin 4 mg/kg daily for four days and monthly subcutaneous givosiran 2.5 mg/kg monthly, a siRNA molecule directed against aminolevulinic acid 1 (ALAS1), the gene implicated in the accumulation of neurotoxic porphyrins in AIP. After beginning her treatment with the hematology and oncology departments one week post discharge, her symptoms have been managed effectively by the treatment plan and regular three-month follow-up. This has enabled her to engage in her college activities without any problems. Additionally, she has not required any more hospital stays.

**Table 3:** Urine porphyrin results

Urine Porphyrin	Lab Values	Reference Range
Random porphobilinogen (mg/g creat)	76.064	<0.22
Uroporphyrin I (mcg/g creat)	7164.3	3.6-21.1
Uroporphyrin III (mcg/g creat)	6982.7	<5.6
Heptacarboxyporphyrin (mcg/g creat)	286	<3.4
Hexacarboxyporphyrin (mcg/g creat)	54.4	<6.3
Pentacarboxyporphyrin (mcg/g creat)	242.5	<4.1
Coporporphyrin I (mcg/g creat)	188.4	6.5-33.2
Coproporphyrin III (mcg/g creat)	846	4.8-88.6
Total porphyrins (mcg/g creat)	15754.3	27-153.6

**Table 4:** Serum porphyrin results

Serum Porphyrin	Lab Values	Reference Range
Plasma total porphyrins (mcg/L)	20.1	1.0-5.6

## Discussion

AIP is a rare metabolic disorder in the porphyria family characterized by an abnormality in heme biosynthesis, leading to the accumulation of toxic precursors in the liver and causing a wide array of symptoms [2,4]. The enzymatic defect in AIP results in a partial deficiency of PBGD, a critical enzyme in the heme biosynthesis pathway. In AIP, the activity of this enzyme is significantly reduced (typically to less than 50% of normal), leading to an accumulation of PBG and ALA in the liver and subsequent spillage into the circulation, urine, and feces [2,4]. The accumulation of these neurotoxic precursors is thought to be responsible for the neurological and abdominal symptoms characteristic of acute attacks [2,4]. The expression of AIP symptoms is often triggered by certain drugs, fasting, infections, alcohol, and hormonal fluctuations [2,4,5]. This disruption can lead to a wide range of neurological symptoms, including abdominal pain (due to autonomic neuropathy), peripheral neuropathy, seizures, and mental status changes [2,5].

Over 400 mutations have been identified for the HMBS gene, contributing to the heterogeneity in clinical presentation and severity of the disease [6]. Despite the relatively uniform distribution of genetic mutations associated with AIP across genders, clinical expression tends to be more common in women [7]. This phenomenon is often attributed to hormonal factors, particularly the role of progesterone [7]. The role of progesterone in AIP is linked to its potential to exacerbate symptoms. Progesterone, along with other steroid hormones, can induce the hepatic ALAS1, the first enzyme in the heme biosynthesis pathway. This induction increases the demand on the heme biosynthesis pathway, which can lead to the accumulation of porphyrin precursors when the pathway is deficient, as in AIP [6,7].

The clinical presentation of AIP is heterogeneous, with symptoms ranging from mild to life-threatening [1]. The hallmark features of AIP include acute neurovisceral attacks that manifest with a constellation of symptoms [1]. Abdominal pain is the most common presentation, occurring in 8% to 95% of patients [4]. The pain is described as severe, diffuse abdominal pain without physical findings [2]. The pain is typically non-localizing and can mimic surgical emergencies such as acute appendicitis or pancreatitis [2]. Neurological symptoms are also common, including peripheral neuropathy, characterized by weakness, tingling, and pain in the extremities, as was also seen in our case [5]. The patient can also experience episodes of confusion and hallucinations [5]. Acute attacks of AIP can lead to severe chronic neurological complications, including motor neuropathy that may progress to paralysis and respiratory failure if untreated [5]. Psychiatric manifestations, ranging from mild anxiety and depression to severe psychosis, can often precede the diagnosis of AIP or occur concomitantly with other symptoms, as they did in our case [3]. Patients may exhibit signs of autonomic dysfunction, including tachycardia, hypertension, and sweating [6]. The protean and non-specific nature of symptoms associated with AIP often leads to delays in timely diagnosis [3,5].

The initial step in diagnosing AIP involves biochemical testing for the increased excretion of porphyrin precursors in the urine, specifically PBG and ALA [2,4]. Although not specific to AIP, plasma and fecal porphyrin analyses can provide supporting evidence for porphyric syndrome [1]. In our case, both plasma and urine were positive for PBG. The confirmatory test for diagnosing AIP is through genetic testing, which involves identifying mutations in the HMBS gene, which encodes the enzyme HMBS [3,6]. In our case, genetic testing revealed a mutation-positive for the HMBS gene, providing definitive diagnostic confirmation. Finally, given the nonspecific nature of AIP symptoms, part of the diagnostic process involves ruling out other conditions that can present with similar symptoms, including other gastrointestinal disorders, neurological conditions, and psychiatric illnesses [8].

The treatment of AIP focuses on managing acute attacks, preventing triggers, and long-term monitoring to mitigate the risk of recurrent episodes [2,4]. The cornerstone of treating acute attacks in AIP is the administration of intravenous hemin, which suppresses the hepatic ALAS1, thereby reducing the overproduction of porphyrin precursors [2]. High-dose glucose administration is another strategy used in the initial management of acute attacks, particularly in mild cases or while awaiting hemin therapy, exerting a repressive effect on ALAS1 [5]. However, glucose therapy's effectiveness is more variable and less potent than hemin [5]. Recent studies have also explored novel therapeutic approaches, including gene therapy and RNA interference, promising targeted treatments for AIP [9,10]. Long-term management focuses on preventing acute attacks through lifestyle modifications such as a balanced diet with sufficient carbohydrates and avoidance of known precipitating factors, such as certain drugs, alcohol, and fasting [9,10].

## Conclusions

In conclusion, AIP presents a complex clinical challenge requiring high clinical suspicion for effective diagnosis and management. Further research is needed to optimize AIP's long-term management and explore the full potential of emerging therapies.
